# Small molecule inhibition of the CHFR-PARP1 interaction as novel approach to overcome intrinsic taxane resistance in cancer

**DOI:** 10.18632/oncotarget.5040

**Published:** 2015-09-01

**Authors:** Seth A. Brodie, Ge Li, Donald Harvey, Fadlo R. Khuri, Paula M. Vertino, Johann C. Brandes

**Affiliations:** ^1^ Department of Hematology and Medical Oncology, Emory University, Atlanta, GA, USA; ^2^ Department of Radiation Oncology, Emory University, Atlanta, GA, USA; ^3^ Winship Cancer Institute, Emory University, Atlanta, GA, USA; ^4^ Tennessee Oncology, Nashville, TN, USA

**Keywords:** taxanes, chemoresistance, lung cancer, CHFR, mitotic checkpoint

## Abstract

The mitotic checkpoint protein CHFR has emerged as a major mediator of taxane resistance in cancer. Here we show that CHFR's PAR-binding zinc finger domain (PBZ) mediates a protein interaction with poly-ADP ribosylated PARP1 leading to stabilization of CHFR. Disruption of the CHFR-PARP1 interaction through either PARP1 shRNA-mediated knockdown or overexpression of a PBZ domain peptide induces loss of CHFR protein expression. In an attempt to exploit this observation therapeutically, and to develop compounds with synthetic lethality in combination with taxanes, we performed a high-throughput computational screen of 5,256,508 chemical structures against the published crystal structure of the CHFR PBZ domain to identify candidate small molecule CHFR protein-protein interaction inhibitors. The 10 compounds with the best docking scores (< −9.7) were used for further *in vitro* testing. One lead compound in particular, termed ‘A3’, completely disrupted the protein-protein interaction between CHFR and PARP1, resulting in the inhibition of mitotic checkpoint function, and led to therapeutic synergy with docetaxel in cell viability and colony formation assays. In mouse xenografts, i.p. administration of ‘A3’ led to a significant reduction in nuclear CHFR protein expression with a maximal effect 4 hours after administration, confirming relevant pharmacodynamics following the peak of ‘A3’ plasma concentration *in vivo*. Furthermore, combination of A3 and taxane led to significant reduction of implanted tumor size without increase in hematological, hepatic or renal toxicity. These findings provide a proof-of-principle that small molecule inhibition of CHFR PBZ domain interaction is a novel potential therapeutic approach to increase the efficacy of taxane-based chemotherapy in cancer.

## INTRODUCTION

Microtubular-targeted chemotherapy agents such as taxanes are among the most widely prescribed first- and second-line chemotherapy choices for patients with the most common malignancies including lung-, breast-, and prostate cancer. Unfortunately, primary resistance to taxanes is common and poses an important clinical problem. The mitotic checkpoint gene “checkpoint with forkhead and ringfinger domains” (CHFR) has recently emerged as a critical mediator of resistance against microtubular-targeted therapies in various different cancer types including gastric-[1], colon-[2, 3], endometrial-,[4, 5] and lung cancer [6]. Epigenetic silencing of CHFR by promoter hypermethylation or reduced protein expression have been described as predictive biomarkers for taxane sensitivity. We have previously reported that advanced lung cancer patients with CHFR deficient lung cancers have remarkably high clinical benefits rates following taxane-based first line chemotherapy (81% vs 48%, *p* = 0.03) and significantly improved overall-survival (HR = 0.24; 95% CI, 0.1–0.58%; *P* = 0.002) suggesting that in this setting, taxanes can be considered targeted therapy against CHFR-low expressing tumors [6]. CHFR expression is reduced in tumors that are driven by EGFR mutations in exons 19 or 21, but EGFR mutations do not account for all cases of reduced CHFR expression [7].

CHFR is an antephase checkpoint gene that functions to delay cell cycle entry into metaphase in response to mitotic stress [8], allowing for subsequent repair of taxane induced microtubular damage. Cells that are deficient in this checkpoint undergo mitotic catastrophe and apoptosis, explaining the increased sensitivity of CHFR negative tumors towards microtubular targeted therapies.

CHFR has an N-terminal forkhead domain, a RING domain which functions as an E3-ubiqutin ligase, and a cysteine-rich C terminal domain, which mediates interactions with other proteins. CHFR controls the activity of the aurora-kinase A [9] and polo-like kinase 1 [10] and can exclude cyclin B1 from the nucleus [11]. Mice deficient in CHFR develop spontaneous malignancies and are more susceptible to chemical carcinogenesis [9].

Recently, a poly-ADP ribose binding zinc-finger (PBZ) motif was identified in the C-terminal region of CHFR [12], which was shown to mediate a protein-protein interaction with PARP-1. The functional importance of this interaction between PARP1 and CHFR is two-fold: First, it allows CHFR to be recruited to areas of DNA damage, where together with RNF3 it co-facilitates ubiquitination of histone proteins, leading to a more relaxed chromatin pattern thus allowing ATM to initiate a DNA damage response [13, 14]. Secondly, through CHFR-mediated ubiquitination of PARP-1 and its subsequent proteosomal degradation, it acts to remove PARP-1 from damaged chromatin once the DNA repair machinery has been initiated [15]. Mutations in the PBZ domain lead to a loss of CHFR's mitotic checkpoint function, even though the role of PARP1 in response to microtubular damage is so far unclear.

Given the facts that reduced CHFR expression or epigenetic silencing is clearly associated with better clinical responses and even more importantly, improved overall survival following taxane based therapy in a variety of cancers and that the CHFR's PBZ domain is essential for its checkpoint function, we hypothesized that targeting the protein-protein interactions mediated by the CHFR PBZ domain could be exploited as a strategy to increase taxane sensitivity in tumors with high CHFR expression. The goal of this study was to indentify and characterize small molecule inhibitors against the CHFR PDZ domain.

## RESULTS

### PBZ mutant CHFR fails to induce taxane resistance in CHFR deficient NSCLC cell lines

**Figure 1:**
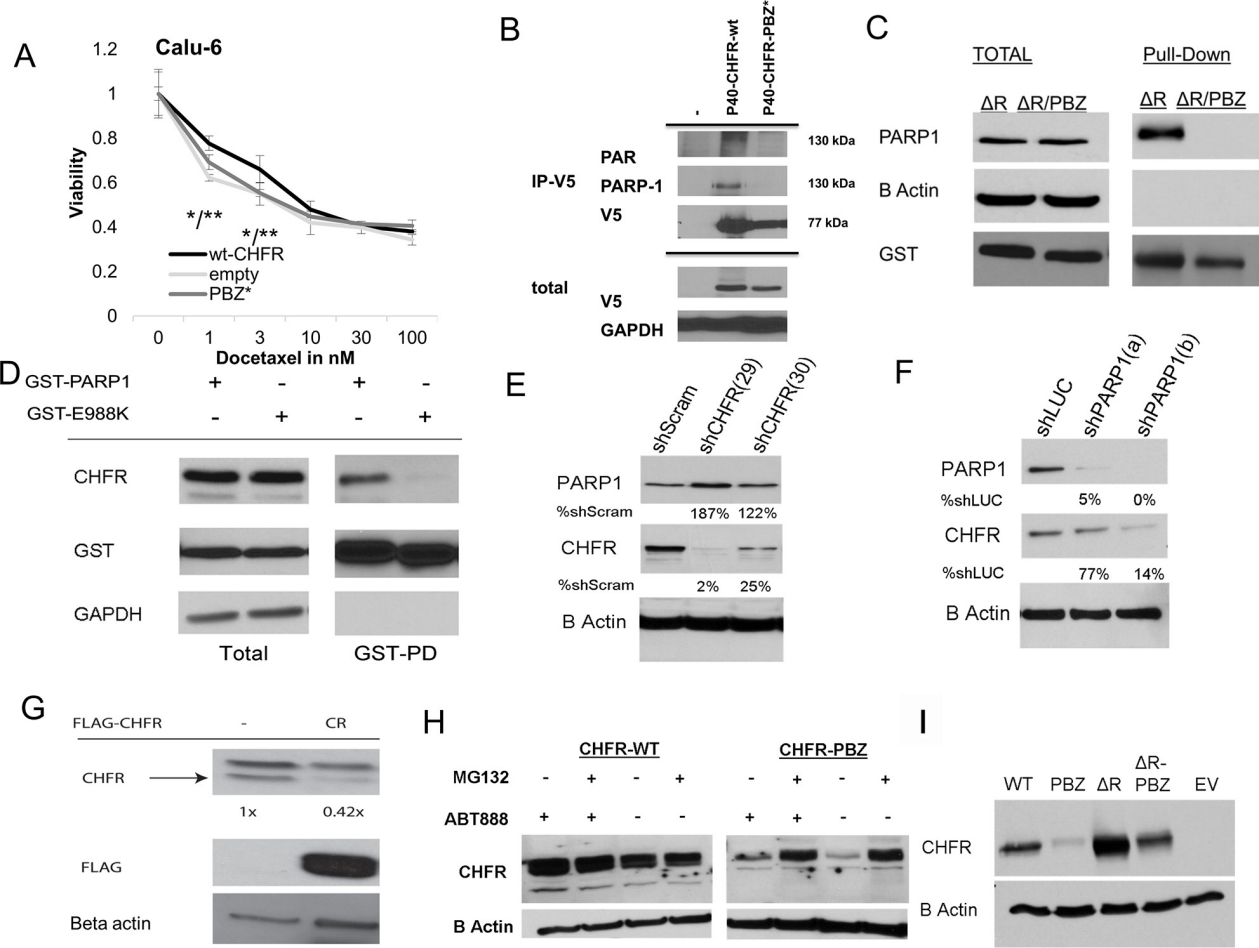
**A.** pDEST40-wt-CHFR, pDEST40-CHFR-PBZ* (mutated PBZ domain) and empty pDEST40 vector were transfected into CHFR deficient Calu-6 NSCLC cells. The effect of Docetaxel after 72 hours was established by XTT assay. Stable transfection of wt-CHFR increases taxane resistance over empty vector and over CHFR-PBZ* (*p < 0.05 for CHFR-wt vs empty vector; **p < 0.05 for CHFR-wt vs CHFR-PBZ). **B.** pDEST40-wt-CHFR and p40-CHFR-PBZ* was transfected into HEK293 cells. After immunoprecipitation with an anti-V5 antibody, an interaction only between wt-CHFR and PAR and PARP1 was demonstrated **C.** pDEST27-ΔR-CHFR and pDEST27-ΔR-CHFR-PBZ* were stably transfected into HEK293 cells. Deletion of the RING domain stabilized and equalized protein levels of both constructs. After affinity purification with a gluthatione resin, only the CHFR mutant with an intact PBZ domain (−ΔR-CHFR) interacted with PARP1. Mutation in the PBZ domain (−ΔR-CHFR-PBZ*) completely abrogated this interaction. **D.** pDEST27-PARP1 and enzymatically deficient pDEST27-PARP1-E988K were transfected into HEK293 cells. After glutathione-affinity purification, an interaction with CHFR was only demonstrated for the catalytically intact wt-PARP1, suggesting that auto-parylation of PARP1 is required for the interaction with CHFR. **E.** stable shRNA knockdown of CHFR in A549 cells leads to a reduction in PARP1 protein levels compared to scrambled shRNA; (number indicates stable cell line annotation) **F.** stable shRNA knockdown of PARP1 in A549 cells leads to a reduction in CHFR protein levels compared to luciferase targeted shRNA (number indicates stable cell line annotation) **G.** Native CHFR protein levels in A549 cells are reduced by overexpression of a FLAG tagged PBZ-domain peptide as competitor for parylated-PARP1. **H.** While stable transfection of CHFR-PBZ* in Calu-6 cells leads to only low levels of expression, proteasomal inhibition with MG-132 increases CHFR-PBZ* protein levels. Treatment with the PARP-inhibitor ABT-888 does not have a significant impact on either wt-CHFR or CHFR-PBZ* levels. **I.** A mutation in the RING domain (CHFR-ΔR-PBZ*) leads to protein levels comparable to those of wt-CHFR, indicating that auto-ubiquitination and –degradation of CHFR are responsible for the low expression of CHFR-PBZ*EV, empty vector; PBZ*, PBZ mutated CHFR; ΔR, Ring domain mutated CHFR.

Transfection of wt-CHFR into CHFR deficient cells has previously been shown to restore the antephase checkpoint leading to a pre-mitotic cell cycle arrest after taxane challenge and ultimately to confer de-novo resistance to taxanes [8]. In Hela cells, it was suggested that full length, but not PBZ-mutant CHFR has similar cell cycle effects [12]. To determine the functional relevance of the PBZ domain on taxane resistance in NSCLC, we transfected CHFR deficient CALU-6 cells either with full-length CHFR (pDEST40-wt-CHFR) or PBZ mutant CHFR (pDEST40-CHFR-PBZ*). Cell viability assays showed that only transfection of wt-CHFR confers resistance to taxanes when compared to both transfection of empty vector or the PBZ mutant variant (Figure 1A). These findings highlight the importance of an intact PBZ domain for an intact checkpoint function and CHFR mediated taxane resistance, since the CHFR-PBZ* construct did not affect taxane sensitivity compared to empty vector. CHFR protein levels are shown in Supplementary Figure S1A.

### The CHFR PBZ domain mediates interactions with parylated PARP1

To determine possible interactions between CHFR and other proteins that depend on an intact PBZ domain, we transfected the empty pDEST40 vector, pDEST40-wt-CHFR or pDEST40-CHFR-PBZ* vectors into HEK293 cells and performed immunoprecipitation for V5-tagged CHFR. Western blot analysis for poly ADP ribosylated (PAR) proteins revealed one prominent band at ∼130kDA interacting only with wt-CHFR but not CHFR-PBZ*, suggesting an interaction with one major species of PARylated protein. Given that the molecular weight of parylated PARP1 is approximately 130 kDA, we performed a western blot for PARP1, which confirmed an interaction between CHFR and PARP1 (Figure 1B). Since protein expression of a PBZ mutant CHFR construct is unstable, we transfected either wt-CHFR or CHFR-PBZ constructs with an additional deletion of the RING domain. These constructs show preserved and equal protein expression. Mutation of the CHFR-PBZ domain abolished the interaction between CHFR and PARP1, indicating that the interaction requires an intact PBZ domain (Figure 1C). The reciprocal interaction between PARP-1 and CHFR was also detected in HEK 293 cells expressing GST tagged wt-PARP-1 (Figure 1D). To determine if the interaction between CHFR and PARP1 is parylation dependent or independent, we generated a catalytically dead mutant of PARP1 (E988K), which completely lacks the ability to synthesize PAR [20]. Only wt-PARP1 showed an interaction with CHFR (Figure 1D), suggesting that the interaction between these two proteins is parylation-dependent. Taken together, these data indicate that CHFR preferentially interacts with the parylated form of PARP-1, and that this interaction is mediated by the CHFR PBZ domain.

### The interaction with PARP1 stabilizes CHFR protein

We next determined the impact of CHFR on PARP1 stability and vice versa. shRNAs were used to generate stable knockdowns in HEK293 cells. CHFR knockdown led to an increase in PARP-1 protein expression (Figure 1E). This phenomenon has previously been attributed to the decrease in proteasomal degradation of PARP1. Conversely and interestingly, PARP1 knockdown led to a significant reduction in CHFR protein expression (Figure 1F). These findings suggest that the interaction with PARP1 stabilizes the CHFR protein, possibly by protecting it from degradation. If the interaction with PARP1 protects CHFR from degradation, then CHFR protein levels should be reduced by competing for this interaction. To test this hypothesis, we overexpressed a Flag-tagged PBZ domain alone in HEK293 cells. Indeed, CHFR protein levels were reduced in cells expressing the PBZ domain as compared to those transfected with empty vector (Figure 1G), supporting the above hypothesis. Together, these results demonstrate that CHFR protein is indeed stabilized by its interaction with PARP1 which is mediated by CHFR's PBZ domain.

### CHFR degradation in PBZ mutant CHFR is mediated by autoubiquitination

Regulation of CHFR protein levels has previously been shown to be at least in part dependent on autoubiquitination mediated by its RING domain [21]. To determine if disruption of the CHFR-PARP1 interaction forces CHFR degradation by auto-ubiquitination and subsequent proteasomal degradation, we expressed wild-type and the CHFR-PBZ mutant in the presence and absence of the proteasome inhibitor MG-132 (Figure 1H) or after the additional deletion of the RING domain (Figure 1I) in Calu-6 cells. While the CHFR PBZ mutant protein is only expressed at very low levels, both inhibition of the proteasome or deletion of the RING domain restored PBZ mutated CHFR expression to levels that were comparable to those of the wt-CHFR. These data are consistent with the interpretation that disruption of the CHFR-PARP interaction results in auto-ubiquitination and degradation of CHFR. Pharmacologic inhibition of PARP1's polymerase activity by the PARP-inhibitor ABT-888 did not result in altered CHFR protein levels (Figure 1H).

### The CHFR-PARP1 interaction is cell cycle dependent and enhanced by docataxel induced mitotic stress

**Figure 2:**
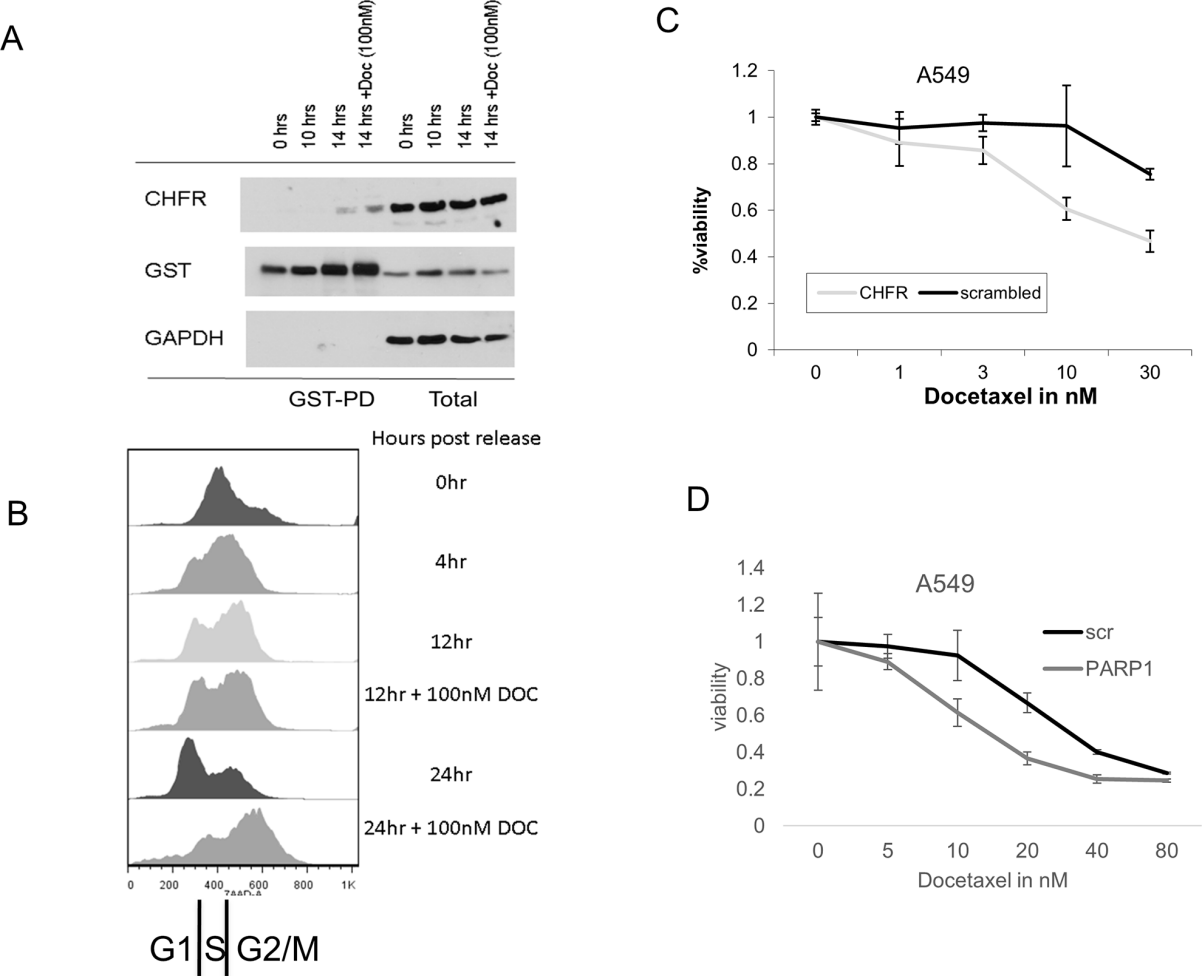
**A.** and **B.** In aphidocholin synchronized A549 cells, an interaction between GST-tagged PARP1 and CHFR is predominantly observed in the G2/M phase of the cell cycle. Induction of microtubular damage with docetaxel further enhances this interaction, suggesting a central role in mediating the antephase checkpoint. Stable knockdown of both **C.** CHFR and **D.** PARP1 sensitizes stably transfected A549 cells to docetaxel compared to scrambled control shRNA.

We next sought to determine the functional significance of the interaction between CHFR and PARP1 in antephase checkpoint control and taxane sensitivity, HEK293 cells were transfected with a GST-tagged PARP1, synchronized with aphidocholin, and the interaction between GST-tagged PARP1 and CHFR was analyzed at various stages of the cell cycle (Figure 2A, 2B, Supplementary Figure S3). The interaction between PARP1 and CHFR was limited to the G2/M phase of the cell cycle and was further enhanced by additional exposure to mitotic stress, suggesting a specific role of this interaction in the control of mitotic entry. To determine the effect on taxane sensitivity, we performed XTT assays in A549 cells stably transfected with either shRNAs specifically targeting CHFR or PARP1 or control shRNAs (scrambled or luciferase targeting respectively, (Supplementary Figure S1B and S1C)). As expected, CHFR-deficient A549 cells showed an increased sensitivity to docetaxel relative to A549 cells expressing a scrambled control (Figure 2C). Interestingly, PARP1 deficient cells also showed increased sensitivity to docetaxel (Figure 2D), likely due to the reduction in CHFR expression that accompanies PARP1 knockdown (Figure 1G). These findings show that not only CHFR is required for a functional mitotic checkpoint, but that additionally PARP1 is also an important mediator of taxane resistance.

### PARP-inhibition fails to induce synergistic cytotoxicity in CHFR expressing lung cancer cell lines

Hypothesizing that PARP-inhibition could induce synergistic cytotoxicity in CHFR expressing lung cancer cell lines, we performed cytotoxicity assays in seven lung cancer cell lines (A549, EKVX, H596, H522, Hop-62, H460 and H2023) using docetaxel and the PARP-inhibitor ABT-888 either alone or in combination. Synergy was not observed in any of the cell lines. Also, combination of docetaxel with a dose of ABT-888 (20 uM), which achieves PARP inhibition *in vitro*, did not lead to enhanced cytostasis (data not shown). *In vitro*, ABT-888 did not reduce CHFR protein expression (Figure 1H) nor did it disrupt the interaction between CHFR and PARP-1 (not shown), suggesting that PARP-1 inhibition is insufficient to force CHFR into auto-degradation. We therefore hypothesized that the development of a pharmacologic strategy that mimics the effects of a PBZ domain mutation and completely inhibits the binding of parylated proteins in the PBZ domain may be superior to PARP-1 inhibition in the sensitization against taxanes.

### High-throughput computational screening identifies a small molecule inhibitor of the CHFR/PARP1 interaction

**Figure 3:**
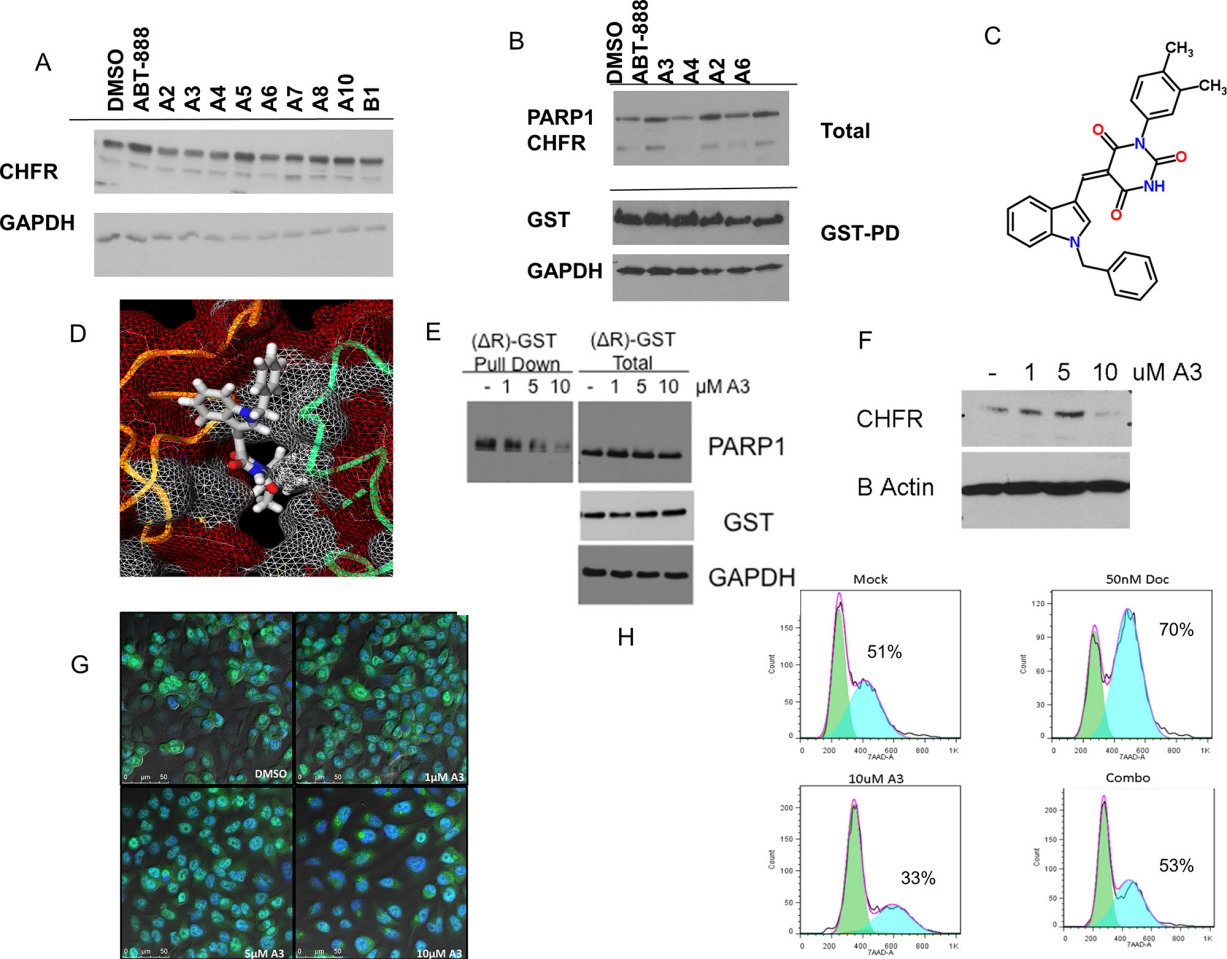
A high-throughput computational screen of 5,256,508 chemical structures against the published crystal structure of CHFR's PBZ domain was performed The 10 compounds with the highest docking scores were tested for their ability to regulate CHFR expression levels. **A.** Several compounds, termed ‘A2’,‘A3’, ‘A4’ and ‘A6’ reduced CHFR expression after 24 hours at 10 uM in A549 cells. **B.** Hek293 cells with stable expression of pDEST27-PARP1, were treated with either DMSO or ‘A3’ at 10 uM, or ABT-888 10 uM for 16 hours and subjected to gluthatione affinity purification for GST tagged PARP1. Only A3 completely inhibited an interaction between PARP1 and CHFR. Chemical structure and predicted binding into the PBZ-domain pocket are shown in **C.** and **D. E.** HEK293 cells were stably transfected with the pDEST27—ΔR-CHFR construct. Deletion of the RING domain in this construct leads to both stable CHFR and PARP1 expression that is unaltered by treatment with increasing concentrations of ‘A3’ for 16 hours. Nonetheless, the interaction between glutathione-affinity purified CHFR-ΔR and PARP1 was decreased by ‘A3’ in a dose dependent fashion. **F.** A549 nuclear extracts treated with vehicle (dmso) or indicated A3 concentration for 16 hrs, CHFR is lost from the nuclear extract at 10 uM A3 **G.** A549 cells were incubated for 16.5 hrs with MG132 and indicated A3 concentration for 16 hrs. At 10 uM A3 concentration CHFR staining is diminished in the nucleus Cells are stained with CHFR(488 green) and Nuclear (DAPI blue) **H.** Co-exposure of unsynchronized cells to docetaxel and ‘A3’ for 24 hours prevents the docetaxel induced cell cycle arrest, suggesting functional disruption of the antephase checkpoint.

In an attempt to generate possible lead compounds for the pharmacologic inhibition of the CHFR-PARP1 interaction, we performed a high throughput computational screen of 5,256,508 chemical structures in the MCULE database of purchasable compounds using the AutoDockVina algorithm against the published crystal structure of the CHFR-PBZ domain. The 10 most promising ‘hits’ based on Autodock Vina docking scores (< −9.7) were selected for further characterization (Supplementary Figure S2). One compound (A9) failed to synthesize. We tested the 9 available compounds for their impact on nuclear CHFR levels (Figure 3A). Several compounds, including ‘A2’, ‘A3’, ‘A4’ and ‘A6’ decreased CHFR levels assayed by western blotting of whole cell extracts. We determined the impact of the above referenced compounds on the PARP1/CHFR interaction. HEK293 cells were transfected with GST tagged PARP1 and the interaction with CHFR determined in pull down assays for those 4 compounds. Only ‘A3’ completely prevented the PARP1-CHFR interaction at 10 uM concentration (Figure 3B). Further studies therefore focused on ‘A3’. Chemical structures are shown in Figure 3C and 3D. In contrast to treatment with ABT-888, ‘A3’ completely disrupted the interaction between GST-tagged PARP-1 and CHFR (Figure 3B). Importantly, *In vitro* parylation studies showed that ‘A3’ has no impact on PARP1 auto-parylation, indicating that the disruption of the PARP1/CHFR interaction by ‘A3’ is not dependent on de-parylation of PARP1, but rather the interaction between CHFR and parylated PARP1 (Supplementary Figure S4). Since it is possible that the co-immunoprecipitation between PARP-1 and CHFR after ‘A3’ treatment was negative due to the fact that CHFR was degraded, we repeated the experiment after transfection with the Ring-domain deleted ΔR-CHFR construct, which maintained both stable CHFR and PARP1 levels even after treatment with ‘A3’. However, ‘A3’ treatment resulted in a dose dependent inhibition of the interaction between CHFR and PARP1, suggesting a specific inhibitory effect of ‘A3’ on this protein interaction (Figure 3E). These observations were confirmed by data that show a dose dependent regulation of CHFR protein levels by ‘A3’ (Figure 3F). Interestingly, however, at low concentrations of ‘A3’ an initial increase in CHFR protein levels was observed, followed by complete disappearance of CHFR at the 10 uM concentration. Since CHFR's mitotic checkpoint function is predominantly executed in the nucleus, we next studied the effects of ‘A3’ treatment on nuclear localization of CHFR. ‘A3’ in combination with MG-132 lead to a depletion of nuclear CHFR and accumulation of cytoplasmic CHFR, suggesting the possibility that ‘A3’ may indeed be capable of disrupting the antephase checkpoint (Figure 3G).

Cell cycle arrest at G2/M in response to microtubular damage is a hallmark of a functional antephase checkpoint. To test the functional relevance of our newly discovered CHFR small molecule inhibitor ‘A3’, we exposed unsynchronized A549 cells for 12 hours to docetaxel (50 nM) with or without ‘A3’ (10 uM) and analyzed the cell cycle stages by flow-cytometry (Figure 3H and Supplementary Figure S5). ‘A3’ exposure prevented docetaxel induced cell cycle arrest, suggesting that ‘A3’ functionally disrupted the antephase checkpoint. This is an important finding since it validates the proposed mechanism of action for ‘A3’.

### Exposure to ‘A3’ has synergistic growth inhibitory activity that correlates with CHFR expression

**Figure 4:**
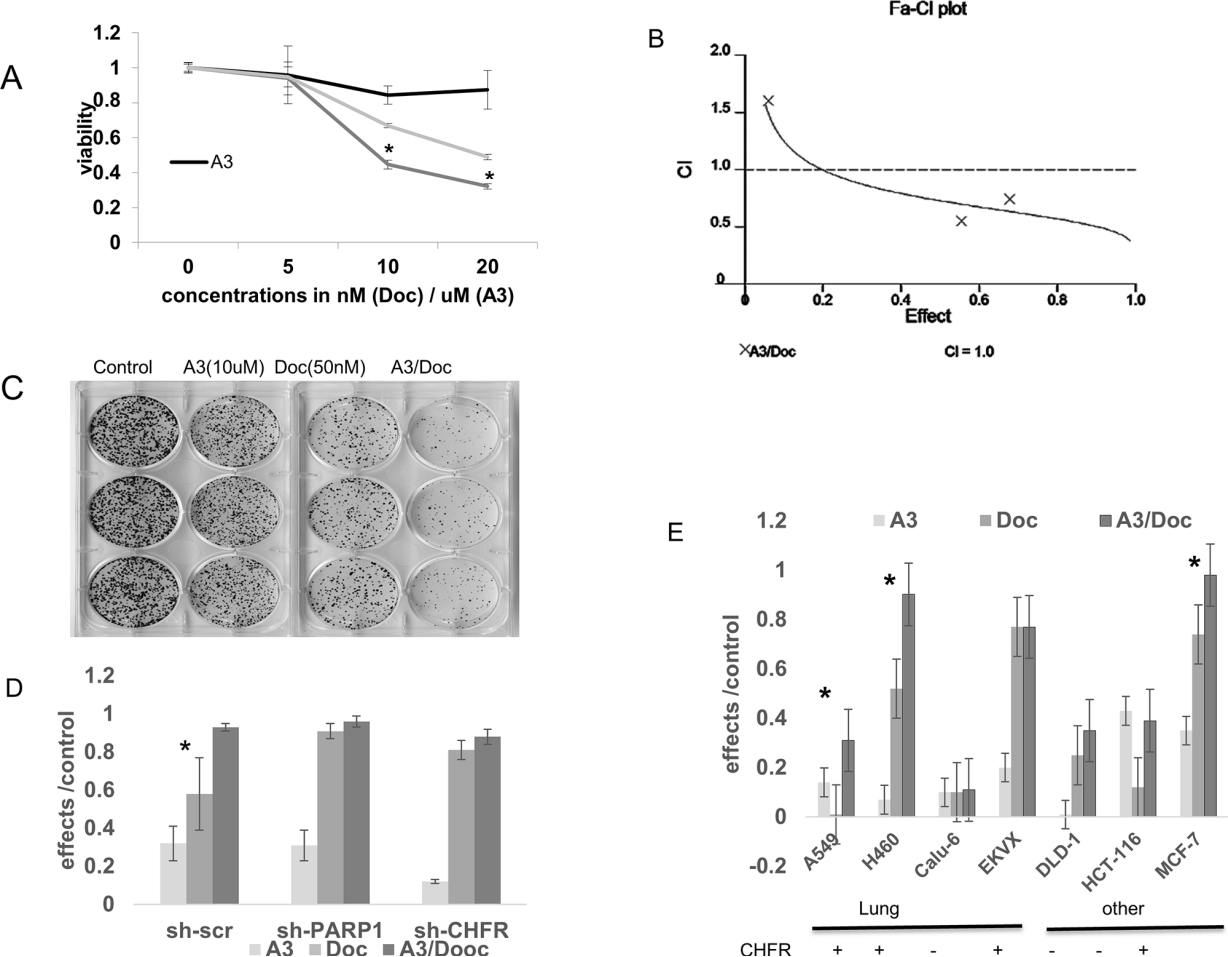
**A.** Cell viability experiments were performed after exposure to ‘A3’ (5–20 uM), docetaxel (5–20 nM) or the combination of both compounds for 72 hours. At the 10 and 20 nM concentrations, there was statistically significantly decreased viability in the combination treated cells. (*= *p* < 0.01). **B.** The combination index was calculated according to the method of Chou-Talaley. ‘A3’ and docetaxel exert synergistic effects at the 10 and 20 uM/nM concentrations B) stable shRNA knockdown of PARP1 or CHFR reverses synergy between A3 and docetaxel in A549 cells in colony formation assays compared with scramble shRNA control (* = *p* < 0.05). **C–E.** The interaction between docetaxel and ‘A3’ was further studied in colony formation assays. Various cell lines of different tissues of origin and with different CHFR expression status were exposed to 1 hour of docetaxel and 72 hours of ‘A3’. Effects were defined as 1- colony count (treatment)/colony count (control). The nature of the interaction between the two compounds was analyzed by the Bliss additivity method. C) A representative example of the colony formation assays is shown in the CHFR expressing cell line H460 where strong synergy between ‘A3’ and docetaxel is observed. For subsequent colony formation assays, cell lines were characterized into CHFR expressing vs. CHFR deficient cell lines by Western-Blot. D) knock-down of either CHFR or PARP1 in A549 cells alleviates the synergy that is observed in A549 cells transfected with scrambled shRNA. (*= synergy) D) synergistic effects of A3 and docetaxel in colony formation assays are mostly observed in CHFR expressing cell lines regardless of tumor type. (*= synergy).

Similarly to the previous experiments in which CHFR knockdown increased taxane sensitivity in A549 cells, ‘A3’ and docetaxel showed synergistic growth inhibitory functions in cell viability assays as demonstrated by a Combination index (CI) < 1 (Figure 4A and 4B). To determine if the synergistic effects of A3 are indeed dependent on an interaction between CHFR and PARP1, we performed colony formation assays in A549 cells transfected with shRNA which was either scrambled or directed against CHFR or PARP1. Synergy between A3 and docetaxel was maintained only in the A549 scramble cell line, but not after CHFR or PARP1 knockdown, suggesting that ‘A3's effects are mainly through its on-target effects on the CHFR-PARP1 interaction (Figure 4D). Finally, we performed colony formation assays on a panel of cell lines of different cancer types such as lung-, breast-, and colon cancer. With the exemption of the EKVX NSCLC cell lines, synergy between ‘A3’ and docetaxel was observed for all other CHFR expressing cell lines regardless of tumor type supporting the possibility that CHFR targeted therapy may have a wide range of possible applications in those cancer types in which taxanes are frequently used (Figure 4C and 4E).

### *In vivo* characterization of A3 levels and activity

**Figure 5:**
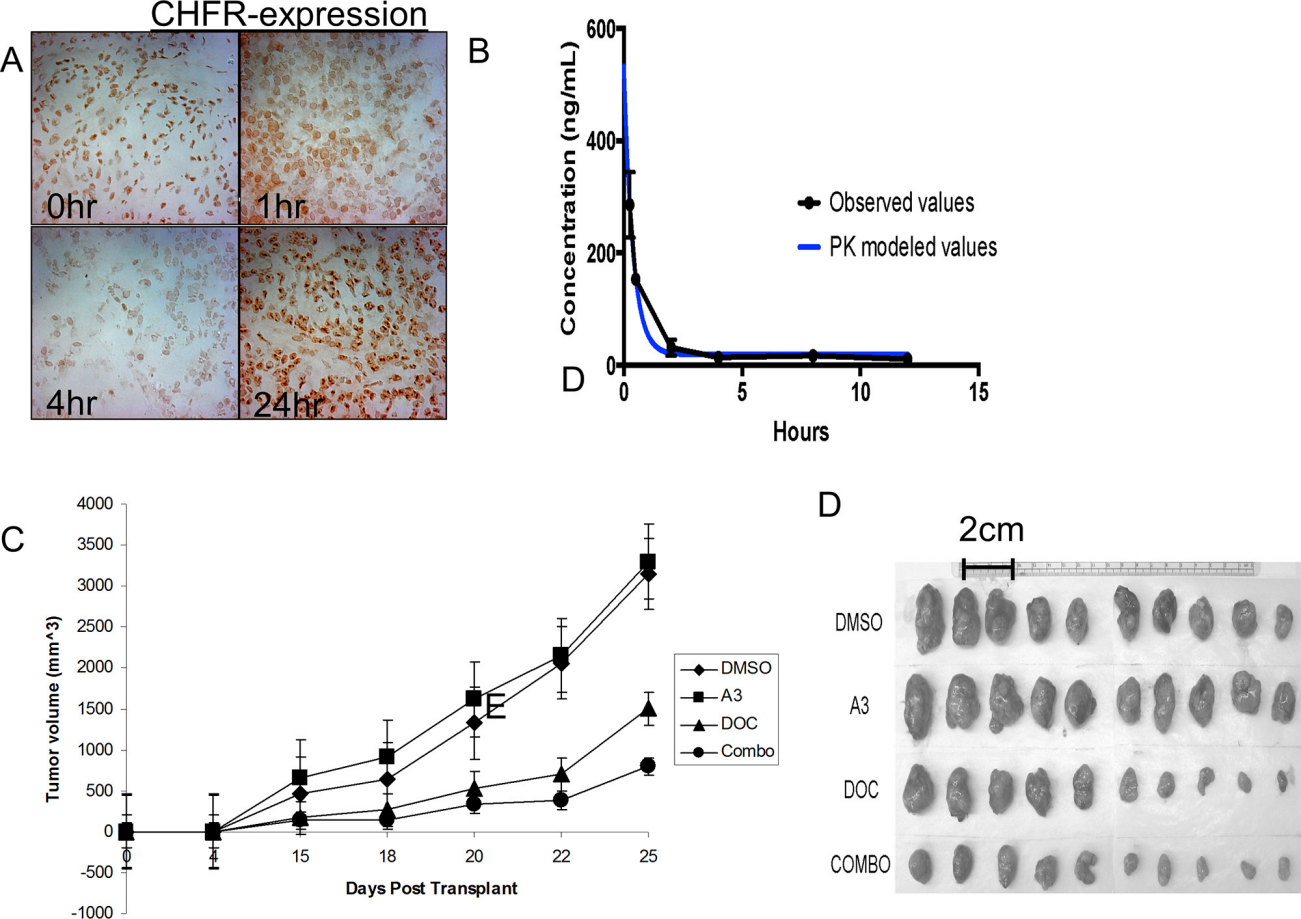
**A.** A 549 cell tumor xenografts in nude mice were analyzed for CHFR expression at various time points after ‘A3’ injection. 4 hrs after i.p. injection a significant reduction in nuclear CHFR staining was observed, indicating a *in vivo* reproducible pharmacodynamic effect of ‘A3’. **B.** Pharmacokinetics of A3 were performed at various time points after iv and oral administration of a single dose of ‘A3’ at 10 mg/kg. **C.** 2.5 × 10^5^ H460 cells were transplanted into the flank of nude mice were. 5 days after transplant, I.P. injection of DMSO, A3, docetaxel, or combination commenced. Tumor size was measured bi-dimensionally and volumes were calculated by volume = (smallest dimension)^2^ × (largest dimension). While ‘A3’ treatment alone did not have a discernable anti-tumor effect, combination treatment with ‘A3’ and docetaxel, proved superior to treatment with docetaxel alone. **D.** Explanted tumors are smallest in mice that were treated with combination therapy.

In the previous section we have demonstrated that ‘A3’ is capable of disrupting the interaction between CHFR and PARP1 *in vitro*, leading to a depletion of CHFR expression and to sensitization towards the cytotoxic effects of taxanes. We next examined the impact of ‘A3’ administration on lung tumor xenografts. A549 NSCLC cells were injected into the flank of nude mice and allowed to establish for approximately 14 days. Once tumors had reached a volume about 100 mm3, mice received a single ip injection of ‘A3’ at 10 mg/kg. Mice were then euthanized at 1,2,4 and 24 hours after and CHFR in the xenografts was analyzed by immunohistochemistry (Figure 5A). Significant suppression of nuclear CHFR expression was observed after 4 hours, but was restored to baseline levels at the 24 hr time point.

We next sought to characterize the *in vivo* properties of ‘A3’. We first determined the pharmacokinetics of ‘A3’ after a single oral (25 mg/kg) and intravenous (10 mg/kg) dose of in CD1 mice. Plasma was obtained at pre-specified time points (15 min to 24 hours) and ‘A3’ concentrations were determined by LC/MS/MS. These data indicated that ‘A3’ was rapidly and widely distributed following intravenous dosing, and the relative bioavailability of ‘A3’ from dose normalized AUC values was calculated as 19.5% (Figure 5B). Oral bioavailability of ‘A3’ was insignificant. The time-point of maximal CHFR suppression at 4 hrs follow the peak in A3 plasma levels with some delay.

Based on these pharmacodynamics and pharmacokinetic properties of ‘A3’, we analyzed the *in-vivo* effects of ‘A3’, docetaxel and the combination on tumor growth in mouse xenografts, who were previously injected with the H460 cell line. Pre-treatment with either DMSO or ‘A3’ was given 4 hours prior to docetaxel injection, when CHFR expression levels are expected to be the lowest based on the above pharmacodynamic experiments. ‘A3’ treatment alone did not have a discernable effect on tumor growth when compared to DMSO alone. However, in combination with docetaxel, ‘A3’ significantly decreased tumor size compared to docetaxel alone, demonstrating that pharmacologic targeting of the CHFR/PARP interaction may indeed be a promising strategy to improve taxane sensitivity in lung cancer.

**Figure 6:**
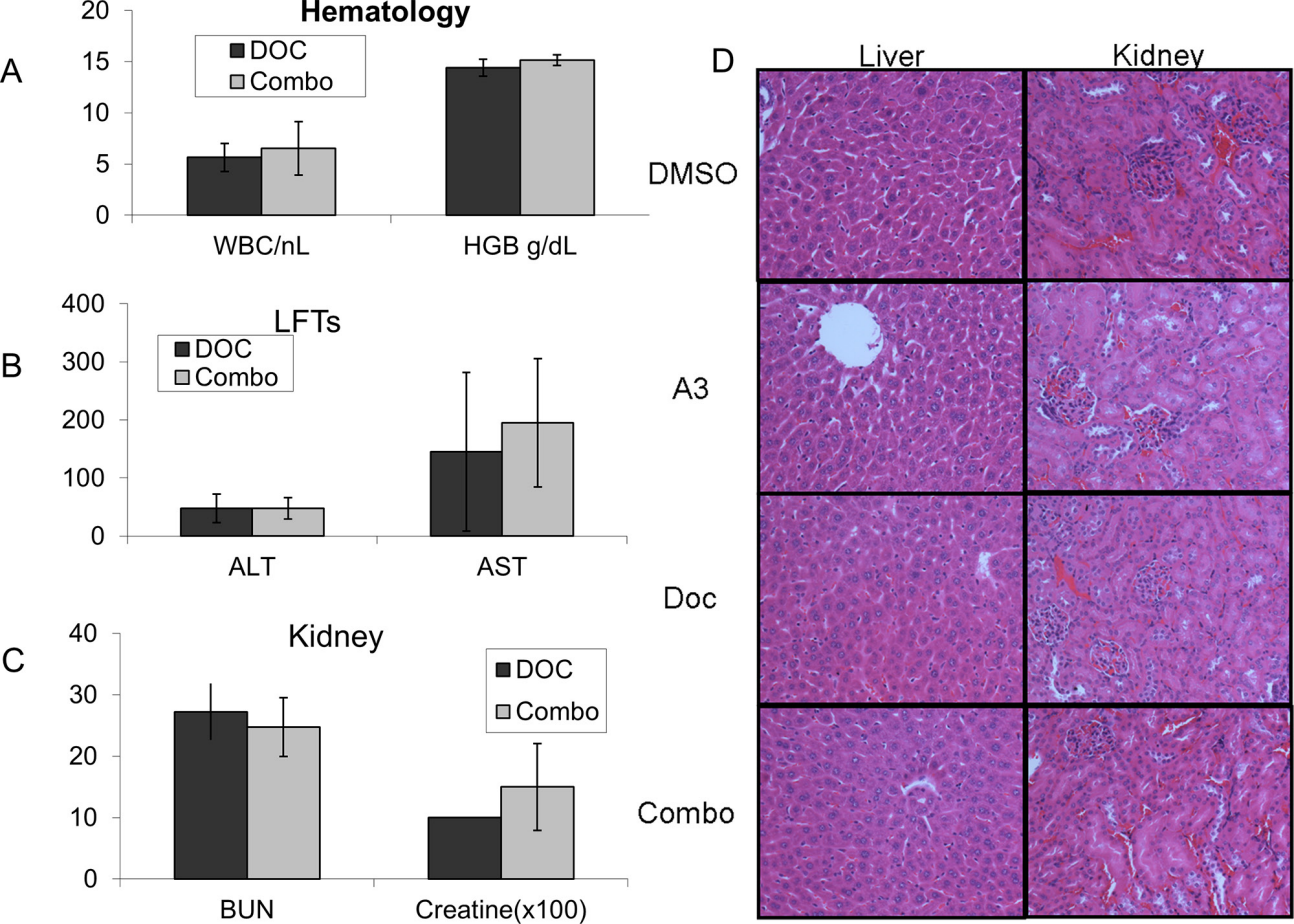
Nude mice were treated with a one-time dose of docetaxel either in combination with ‘A3’ (10 mg/kg), or with equal volumes of DMSO No significant increase in **A.** hematologic **B.** hepatic or **C.** renal toxicity was observed with combination treatment. H&E staining of mouse kidney and liver samples treated with indicated compounds are unremarkable.

Importantly, combination treatment with ‘A3’ was not associated with a significant increase in hematologic (Figure 6A), hepatic (Figure 6B) and renal toxicity (Figure 6C) 10 days after treatment. Representative H&E stained sections of liver and kidneys did not show any toxicity in any of the four treatment arms (Figure 6D).

## DISCUSSION

**Figure 7:**
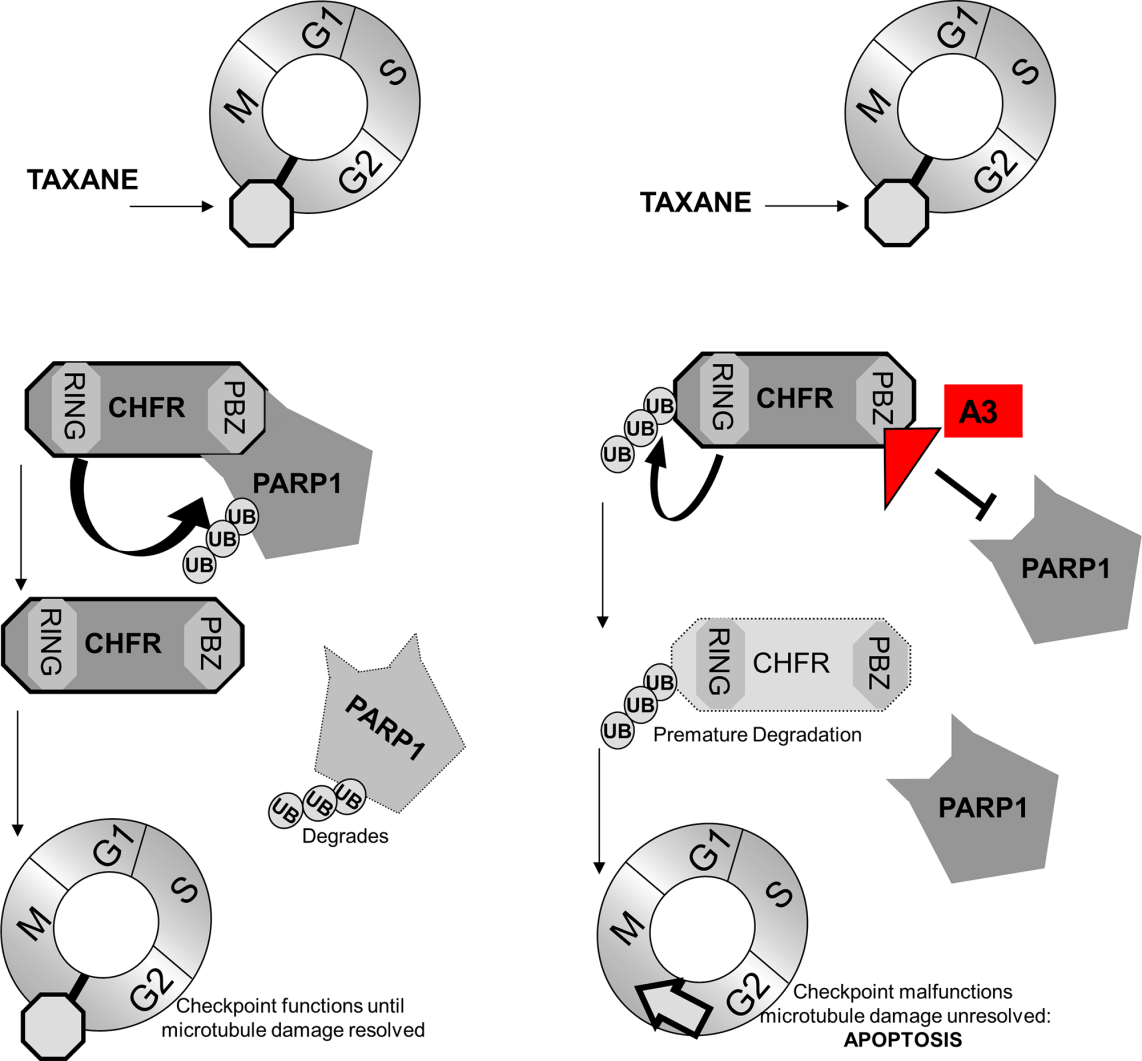
Our findings support a model, by which the interaction between CHFR and PARP1 control expression of both proteins When an interaction is present CHFR targets parylated-PARP1 for proteasomal degradation. PARP1 levels increase with CHFR knockdown. Conversely, a disruption of the interaction between PARP1 and CHFR by the small molecule A3 induces auto-ubiquitination and ultimately destruction of CHFR protein. This in turn leads to disruption of the antephase checkpoint and ultimately mitotic catastrophe in response to taxane challenge.

We have shown in this study, that the interaction between CHFR and parylated PARP1 stabilizes CHFR protein levels. We furthermore show that this interaction is mediated by CHFR's PBZ domain and that its disruption either by mutation of the PBZ domain or PARP1 knockdown leads to auto-ubiquitination and subsequent proteasomal degradation of CHFR. Together with previously published results that reduced CHFR expression in lung cancer is associated with improved survival following platinum taxane based therapy [6] and that taxane sensitivity is increased in gastric [1]-, colon [2]- and cervical cancers [22] in which CHFR is silenced epigenetically, we proposed a model in which pharmacologic inhibition of the CHFR-PARP1 interaction with subsequent loss of CHFR and disruption of antephase checkpoint function helps to overcome intrinsic taxane resistance across a wide spectrum of different tumor types (Figure 7).

This is the first report to demonstrate the feasibility of inhibiting the mitotic checkpoint by targeting the interaction between CHFR and PARP1 with a small molecule. Our data provide several lines of evidence to demonstrate specific target inhibition: First, we show that A3 inhibits the biochemical interaction between PARP1 and CHFR. Second, we find that ‘A3’ treatment leads to the functional disruption of the docataxel-induced mitotic checkpoint, a point in the cell cycle in which the CHFR-PARP1 interaction is greatest. Third, we establish that ‘A3’ synergizes with docetaxel only in CHFR expressing cell lines. Finally, we demonstrate that ‘A3’ administration results in a pharmacodynamic reduction in CHFR expression *in vivo* in human tumor xenograft models.

Our data show clearly that unlike ‘A3’, the PARP1 inhibitor ABT-888 is not capable of disrupting the interaction between CHFR and PARP1. This is surprising since the enzymatically inactive PARP1-E988K mutant does not interact with CHFR. This observation may be explained by the fact that pharmacologic PARP1 inhibition prevents previously un-parylated PARP-1 from autoparylation and may prevent a future interaction with CHFR. Previously parylated PARP-1 however may remain bound to CHFR and PARP inhibition unlike direct small molecule targeting of the PBZ domain will do nothing to force it out of this interaction.

The finding that CHFR protein levels initially increase at low concentrations of ‘A3’ is of potential relevance. The exact explanation for this observation is not entirely clear, but it is interesting to note that this finding was only observed in experiments where CHFR was amendable to proteasomal degradation. Moreover, we did also observe that ‘A3’ exposure enhanced cell proliferation in several lung cancer cell lines including A549 cells. Since CHFR protein is stabilized in G2, we hypothesize that two competing mechanisms are involved in response to ‘A3’: At lower concentrations, a cell cycle mediated increased interaction with PARP-1 may protect CHFR from proteasomal degradation, while at higher concentrations, ‘A3’ completely disrupts the interaction between CHFR and PARP1 leading to loss of CHFR protein expression.

In the development of targeted therapies several aspects deserve consideration: First of all, the functional importance of the molecular target itself needs to be firmly established in order to avoid failure at the clinical stage. The wealth of functional and preclinical data about the role of CHFR in the control of the mitotic checkpoint, as well as the clinical data showing improved response and survival following taxane based therapy in patients with reduced or epigenetically silenced CHFR expression firmly establish CHFR as a promising molecular target in cancer. Our data here show that the pharmacologic targeting of CHFR with a small molecule inhibitor indeed yields synergistic growth inhibitory effects in combination with taxanes.

A second concern is the role of the molecular target in the prevention of treatment related toxicities. In the clinical setting the two major dose limiting toxicities of taxanes are neuropathy and hematotoxicity. If CHFR was integrally involved in preventing these, targeted therapy against CHFR in combination with taxanes could be doomed due to unacceptable toxicities, even if it produced a synergistic anticancer effect. Our data clearly demonstrate that ‘A3’ in combination with docetaxel does not significantly increase hematologic, hepatic or renal toxicities when compared to docetaxel alone. We speculate that hematopoietic precursors may be less susceptible to pharmacologic CHFR inhibition than epithelial or tumor tissues based on analysis of CHFR expression in the Tissue-specific Gene Expression and Regulation (TiGER) database, which reveals that CHFR expression in bone marrow is significantly lower than in epithelial tissues [23]. Taxane induced neurotoxicity is mostly mediated by interference with of the axonal transport, which in turn results in demyelination and axonal degeneration. Since this is a cell cycle independent process it is unlikely that CHFR has a protective function against taxane induced neuropathy.

The third area of concern in successful drug development pertains to the chemical and biochemical properties of the lead compound itself. Our data suggest specificity of ‘A3’ towards CHFR. Its pharmacodynamic properties are promising, but structural variations will be necessary to further improve binding characteristics, bioavailability and elimination half life, while maintaining specificity. Lead optimization should be undertaken before larger scale co-clinical trials in xenografts are performed to assess *in vivo* synergy and toxicity in combination with docetaxel.

In summary, our study represents a novel paradigm to improve taxane sensitivity in cancer therapy by targeting a central mediator of taxane resistance. This study proves the feasibility and specificity of this approach. Refinements in the chemical structure of ‘A3’ will be necessary prior to more extensive *in vivo* studies.

## MATERIALS AND METHODS

### Constructs and transfection

Full length CHFR cDNAs was obtained in pENTR vector from Life Technologies (Grand Island, NY). Full length PARP-1 was cloned by RT-PCR from cDNA obtained from immortalized bronchial epithelial cells (HBEC-3KT) and cloned into pENTR vectors (Life Technology, Grand Island, NY). The following mutants were generated using the Quickchange mutagenesis kit (Agilent Technologies, Santa Clara, CA): 1) CHFR-PBZ* (C623A C629A), 2) CHFR-ΔR (del 305-351), 3) CHFR-ΔR-PBZ*, 4) PARP1-E988K. 5) A FLAG-tagged CR-PBZ-peptide (del 1-406) of CHFR was PCR generated. Using Gateway recombinase kits (Life Technologies, Grand Island, NY), the individual cDNAs were inserted into pDEST26, pDEST27, pDEST40 and pDEST51 vectors, respectively. shRNA against CHFR or scrambled sequence control in pGFP-V-RS vectors were obtained from Origene (Rockville, MD), while shRNA against PARP-1 and luciferase controls were a gift from Dr. Kraus (UT Southwestern). Vectors were transfected using Lipofectamine-2000 (Life Technologies, Grand Island, NY) and stably transfected clones were selected after treatment with G418 or blasticydine or puromycin.

### Cell lines

Cell lines were either purchased directly from ATCC (Manassas, VA) (A549 cells). Other cell lines were a gift from Dr. Vertino. These lines were genotypically validated by STR analysis (Biosynthesis Inc.; Lewisville, TX). All cell lines were cultured in the appropriate media, supplemented with 10% FCS.

### Cell viability assay

2,000 cells/well were seeded in 96 well flat bottom plates. 24 hours later, cells were exposed to either docetaxel alone (1 nM–1000 nM), ‘A3’ alone (1 uM–100 uM) or a combination of those. Mock treated wells received DMSO only. After 48–72 hours, cell viability was analyzed using XTT reagent (Trevigen, Gaithersburg, MD) as colorimetric method. Absorbance was read at 488 nM on a plate reader. Results were plotted using Prism5 software (GraphPad, LaJolla, CA).

### Colony formation assay

Cell lines were seeded at a density of 1,000 cells /well into 6 well dishes. After 24 hrs. cells were treated with either docetaxel (between 50–100 nM) alone or in combination with A3 (10 uM) for 1 hour. After this media was removed, cells were washed in PBS x1 and fresh media was added back. In A3 treated cells, A3 was added back for a total exposure of 72 hours. Equal concentrations of DMSO were added as mock where appropriate. When colonies were visible with the naked eye, cells were fixed in 4% formaldehyde, stained with 0.5% crystal violet and image particles were counted using the ImageJ software.

### Analysis of combination-drug effects

The effects of combined exposure to docetaxel and A3 was compared to single drug exposure in both XTT assays and colony formation assays using the Chou-Talaley method [16]. For effects that were statistically significantly different, a combination index (CI) was calculated using CalcuSyn Software (Biosoft, Cambridge, UK), where a CI < 1 indicates synergistic, a CI = 1 additive and a CI > 1 antagonistic drug effects of the combination. Experiments with single drug concentrations were analyzed using the Bliss additivity method [17], where an additive effect is calculated according to the formula C = A + B – A * B. Effects greater than C are considered synergistic, while effects smaller than C are antagonistic.

### Protein precipitations

Cells expressing H6-tagged and GST-tagged CHFR were lysed in NTA lysis buffer (50 mM Sodium Phosphate, 300 mM NaCl, 10 mM Imidizole, 0.05% Tween-20 pH8) containing PMSF, protease and phosphatase inhibitors. After sonication and clarification by centrifugation, CHFR was affinity purified using nickel-nitrilotriacetic acid (NiNTA) magnetic agarose (Qiagen). Cells expressing V5-tagged proteins were lysed in cell lysis buffer (Cell Signaling, cat #: 9803) containing PMSF and complete protease inhibitors. GST-tagged proteins were isolated using a glutathione sepharose resin (GE healthcare) while V5-tagged constructs were immunoprecipitated using V5 antibodies (Invitrogen) and eluted with V5 peptide (Alpha Diagnostic).

### Immunoblotting

Cells were lysed in 1x cell lysis buffer (Cell Signaling), containing Complete protease inhibitor and Phostop (Roche) and 1 mM PMSF. Cells were sonicated briefly and lysates clarified by centrifugation. Following SDS-PAGE and semi-dry transfer the following antibodies were used: CHFR (Sigma), PARP-1 (Calbiochem), PAR (Tulip), GST (Cell Signaling), V5(Life technologies). In addition, Beta Actin and Beta Tubulin (Sigma) GAPDH (Cell Signaling) were used as loading controls depending on the application and molecular weights of target proteins in the experiment.

### Flow cytometry

Cells were synchronized with 5 uM aphidocholine for 24 hrs, released for the indicated time (0, 6, 12, 24 hrs) and fixed in 70% ice cold Ethanol. Cells were stained with 7-Aminoactinomycin D (7AAD) 250 ng/mL in the presence of 100 ug/mL RNase A. Cells were counted on a BD-FACSCANTO II instrument and analyzed on DIVA and FLOW-Jo software.

### High throughput computational screen for small molecule inhibitors of CHFR's PBZ domain

5,256,508 chemical structures of purchasable compounds were screened against the published crystal structure of CHFR's PBZ domain (PDB ID: 2XOC)[18] using Autodock Vina algorithms on http://www.mcule.com [19]. The top 10 compounds with the best docking scores (< −9.7) were selected and ordered for further study.

### *In vivo* pharmacokinetics

Pharmacokinetics of A3 (5 mg/ml in DMSO) after iv (10 mg/kg) and oral (25 mg/kg) administration were performed in 7 week old male CD-1 mice (Charles River, Raleigh, NC) by the Southern Research Institute (Birmingham, AL). Mice were held in standard housing with maximal 3 mice/cage and fed a standard rodent diet (Harlan, Madison, WI). 4 groups of 3 mice were treated both for iv. and oral dosing schedules. Blood was drawn at various time points (group A: 0.083, 1 hrs.; group B: 0.25, 4 hrs.; group C: 0.5, 8 hrs.; group D: 2, 12 hrs.) and analyzed by HPLC/MS/MS. Mean plasma drug concentration versus time data for both IV bolus and oral dosing were analyzed using the sparse sampling function of non-compartmental analysis with Phoenix^®^ WinNonlin^®^ Version 6.4 (Pharsight Corp.; Cary, NC). Parameters including maximum concentration (C_max_), time to maximum concentration (t_max_), half-life (t_1/2_), and area under the concentration-time curve extrapolated to infinity (AUC_0-∞_), among others, were assessed for both routes, and bioavailability was calculated from dose-normalized values.

### *In vivo* pharmacodynamics

All *in vivo* experiments were approved by the Institutional Animal Care and Use Committee (IACUC) of Emory University and performed according to federal guidelines. Mice were housed in pathogen-free animal facilities of the Winship cancer Institute and fed a standard rodent diet with water at libitum. 9 week old female nude mice (Harlan laboratories) were xenografted with either A549 or H460 cell lines. When tumors reached a size of about 100 mm^3^, mice were injected with ‘A3’ intra-peritoneally at a concentration of 10 mg/kg or an equal volume of DMSO. Mice were euthanized at predefined time points after injection (0 h, 1 h, 4 h, 24 h). Tumors were explanted, imbedded in OCT media and immediately snap frozen in liquid nitrogen. Sections of fresh frozen tissue were prepared at 5 uM thickness and stained for CHFR as described in the immunohistochemistry section.

### *In vivo* efficacy

9 week old female nude mice (Harlan laboratories) were xenografted with H460 cells at a concentration of 2.5 × 10^5^ cells. 5 days after inoculation, mice were randomly divided into four groups of 10 mice to receive the following treatment: A) DMSO at 0 h and 2 h B) ‘A3’ (10 mg/kg) at 0 h and 2 h, C) DMSO at 0 h and 2 h and Docetaxel 15 mg/kg at 2 h, D) ‘A3’ (10 mg/kg) at 0 h and 4 h and Docetaxel 15 mg/kg at 4 h. Treatment was given intra-peritoneally every Monday and Thursday for 2 consecutive weeks. Tumor size was measured tri-dimensionally every 2 days using calipers. When tumor burden scores (defined by http://www.iacuc.emory.edu/documents/tumor_burden_scoring.pdf) exceeded 14, euthanasia was performed with CO2 narcosis. Tumors were immediately explanted, photographed, weighted and frozen in liquid nitrogen.

### *In vitro* auto-parylation

PARP1 HSA (trevigen) was incubated in the presence or absence of A3, ABT888, or equal volume of DMSO at indicated concentrations in PARylation buffer (20 mM Tris 7.9, 100 mM NaCl, 10 mM MgCl2, 10 mM ZnCl2, 10% Glycerol, 1 mM DTT, 1 mg/ml ssDNA (Sigma), 300 uM B-NAD (Amresco)) for 30′ at 30 degrees C. Negative control reactions are performed in the absence of B-NAD. 2x SDS PAGE sample buffer stops the reaction and sample is loaded on SDS-PAGE for subsequent western-blotting for PAR and PARP1.

### Immunohistochemistry (IHC)

OCT media imbedded snap frozen tissue was sectioned on a microtome at 5 uM thickness. IHC was performed using the following antibodies and dilutions: CHFR (1:100) (CellSignaling). After washing and scavenging for free peroxidases, biotin conjugated anti-rabbit antibody was used in 1:500 dilution. Subsequent staining by DAB was performed using a Strep-HRP conjugate and DAB staining kit (vector labs). Slides were imaged on a Zeiss Axioplan2 microscope.

### Statistics

Differences between continuous variables were analyzed by Student's *t*-test.

## SUPPLEMENTARY MATERIALS FIGURES



## References

[1] Satoh A, Toyota M, Itoh F, Sasaki Y, Suzuki H, Ogi K (2003). Epigenetic inactivation of CHFR and sensitivity to microtubule inhibitors in gastric cancer. Cancer research.

[2] Pelosof L, Yerram SR, Ahuja N, Delmas A, Danilova L, Herman JG (2014). CHFR silencing or microsatellite instability is associated with increased antitumor activity of docetaxel or gemcitabine in colorectal cancer. International journal of cancer Journal international du cancer.

[3] Brandes JC, van Engeland M, Wouters KA, Weijenberg MP, Herman JG (2005). CHFR promoter hypermethylation in colon cancer correlates with the microsatellite instability phenotype. Carcinogenesis.

[4] Wang X, Yang Y, Xu C, Xiao L, Shen H, Zhang X (2011). CHFR suppression by hypermethylation sensitizes endometrial cancer cells to paclitaxel. Int J Gynecol Cancer.

[5] Yanokura M, Banno K, Kawaguchi M, Hirao N, Hirasawa A, Susumu N (2007). Relationship of aberrant DNA hypermethylation of CHFR with sensitivity to taxanes in endometrial cancer. Oncol Rep.

[6] Pillai RN, Brodie SA, Sica GL, Shaojin Y, Li G, Nickleach DC (2013). CHFR protein expression predicts outcomes to taxane-based first line therapy in metastatic NSCLC. Clinical cancer research : an official journal of the American Association for Cancer Research.

[7] Brodie SA, Li G, Brandes JC (2015). Molecular characteristics of non-small cell lung cancer with reduced CHFR expression in The Cancer Genome Atlas (TCGA) project. Respiratory medicine.

[8] Scolnick DM, Halazonetis TD (2000). Chfr defines a mitotic stress checkpoint that delays entry into metaphase. Nature.

[9] Yu X, Minter-Dykhouse K, Malureanu L, Zhao WM, Zhang D, Merkle CJ (2005). Chfr is required for tumor suppression and Aurora A regulation. Nature genetics.

[10] Kang D, Chen J, Wong J, Fang G (2002). The checkpoint protein Chfr is a ligase that ubiquitinates Plk1 and inhibits Cdc2 at the G2 to M transition. The Journal of cell biology.

[11] Summers MK, Bothos J, Halazonetis TD (2005). The CHFR mitotic checkpoint protein delays cell cycle progression by excluding Cyclin B1 from the nucleus. Oncogene.

[12] Ahel I, Ahel D, Matsusaka T, Clark AJ, Pines J, Boulton SJ (2008). Poly(ADP-ribose)-binding zinc finger motifs in DNA repair/checkpoint proteins. Nature.

[13] Wu J, Chen Y, Lu LY, Wu Y, Paulsen MT, Ljungman M (2011). Chfr and RNF8 synergistically regulate ATM activation. Nat Struct Mol Biol.

[14] Liu C, Wu J, Paudyal SC, You Z, Yu X (2013). CHFR is important for the first wave of ubiquitination at DNA damage sites. Nucleic Acids Res.

[15] Kashima L, Idogawa M, Mita H, Shitashige M, Yamada T, Ogi K (2012). CHFR protein regulates mitotic checkpoint by targeting PARP-1 protein for ubiquitination and degradation. The Journal of biological chemistry.

[16] Chou TC, Talalay P (1984). Quantitative analysis of dose-effect relationships: the combined effects of multiple drugs or enzyme inhibitors. Adv Enzyme Regul.

[17] Borisy AA, Elliott PJ, Hurst NW, Lee MS, Lehar J, Price ER (2003). Systematic discovery of multicomponent therapeutics. Proc Natl Acad Sci U S A.

[18] Oberoi J, Richards MW, Crumpler S, Brown N, Blagg J, Bayliss R (2010). Structural basis of poly(ADP-ribose) recognition by the multizinc binding domain of checkpoint with forkhead-associated and RING Domains (CHFR). J Biol Chem.

[19] Trott O, Olson AJ (2010). AutoDock Vina: improving the speed and accuracy of docking with a new scoring function, efficient optimization, and multithreading. J Comput Chem.

[20] Patel AG, Flatten KS, Schneider PA, Dai NT, McDonald JS, Poirier GG (2012). Enhanced killing of cancer cells by poly(ADP-ribose) polymerase inhibitors and topoisomerase I inhibitors reflects poisoning of both enzymes. The Journal of biological chemistry.

[21] Chaturvedi P, Sudakin V, Bobiak ML, Fisher PW, Mattern MR, Jablonski SA (2002). Chfr regulates a mitotic stress pathway through its RING-finger domain with ubiquitin ligase activity. Cancer research.

[22] Banno K, Yanokura M, Kawaguchi M, Kuwabara Y, Akiyoshi J, Kobayashi Y (2007). Epigenetic inactivation of the CHFR gene in cervical cancer contributes to sensitivity to taxanes. International journal of oncology.

[23] Liu X, Yu X, Zack DJ, Zhu H, Qian J (2008). TiGER: a database for tissue-specific gene expression and regulation. BMC bioinformatics.

