# Supplementary material for: Small molecule inhibition of the CHFR-PARP1 interaction as novel approach to overcome intrinsic taxane resistance in cancer
Source: Oncotarget. 2015 Sep 1;6(31):30773–86. doi: 10.18632/oncotarget.5040 (PMC4741567; doi:10.18632/oncotarget.5040)
Supplement: Supplementary file 1 [file oncotarget-06-30773-s001.pdf]

SUPPLEMENTARY FIGURES

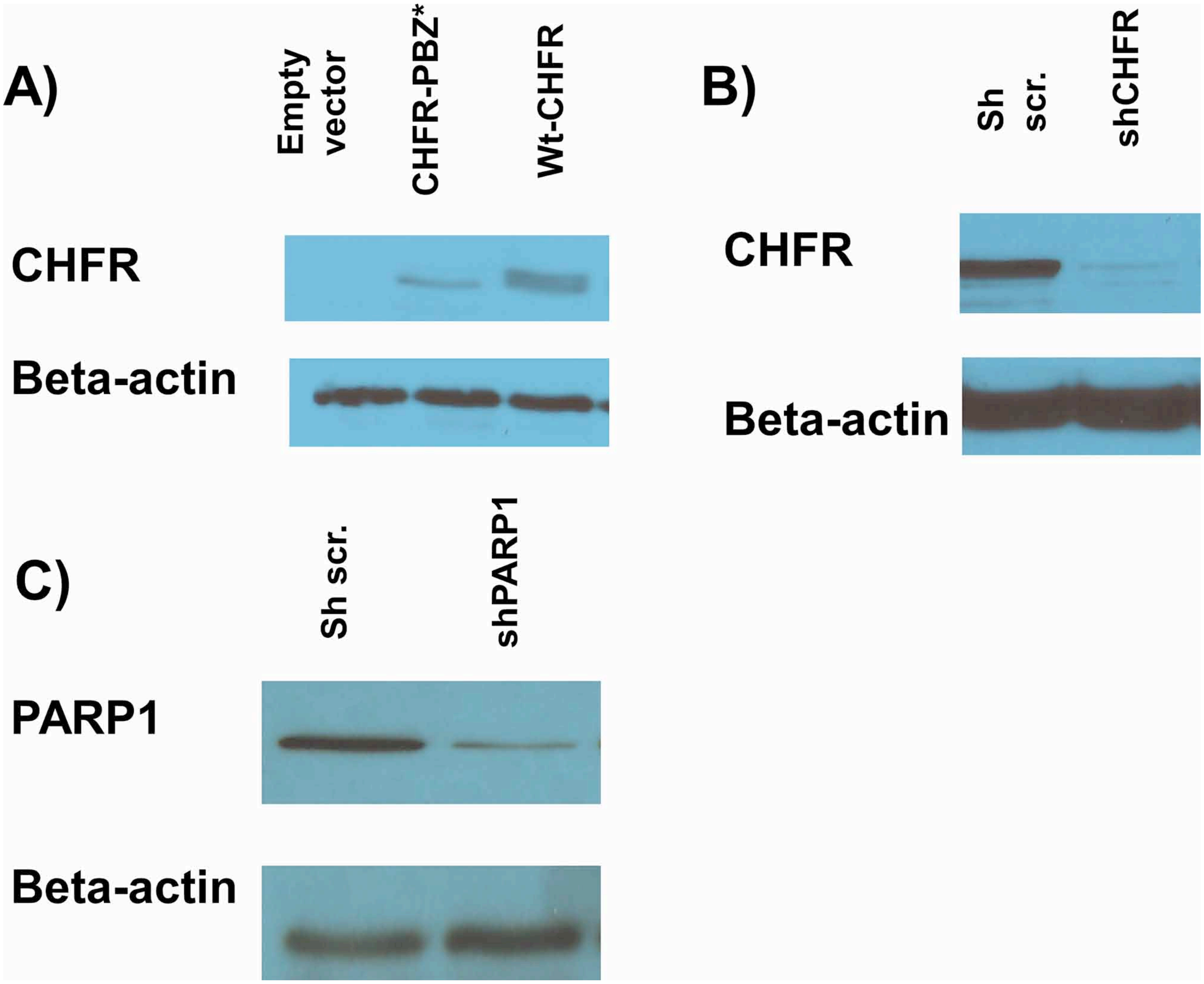

Supplementary Figure S1: Protein expression by Western Blot of stably transfected cell lines used in the cell viability experiments of Figure 1A A., Figure 2C B. and Figure 2D C.

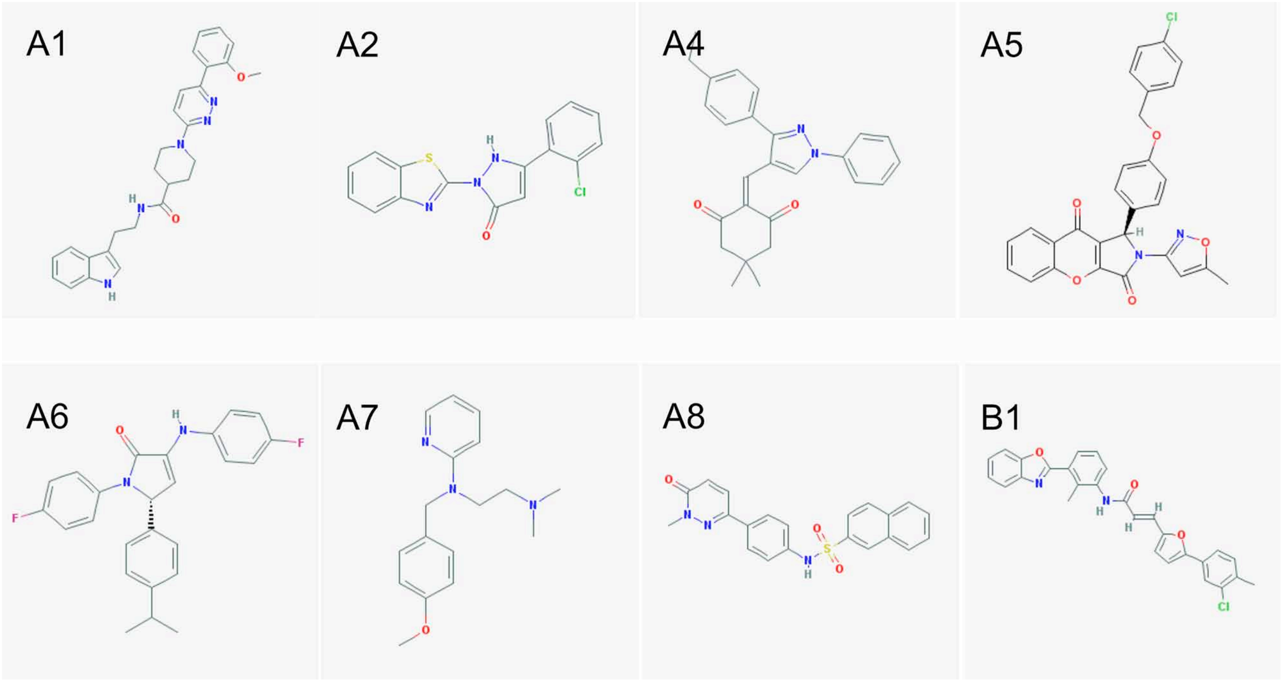

Supplementary Figure S2: Chemical structures of high throughput screening 'hits'.

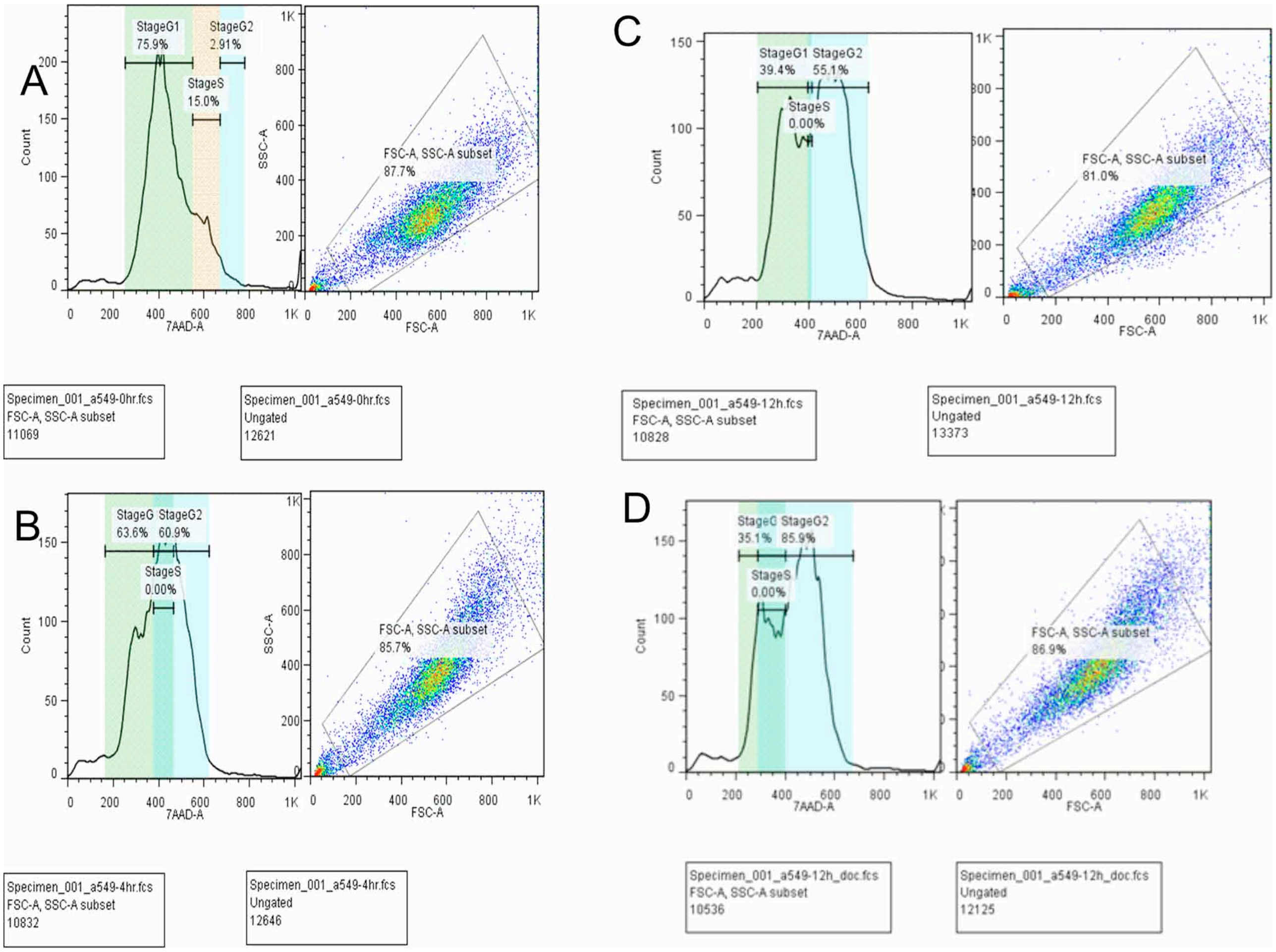

Supplementary Figure S3: Individual analysis of the flow cytometric analysis from Figure 2C.

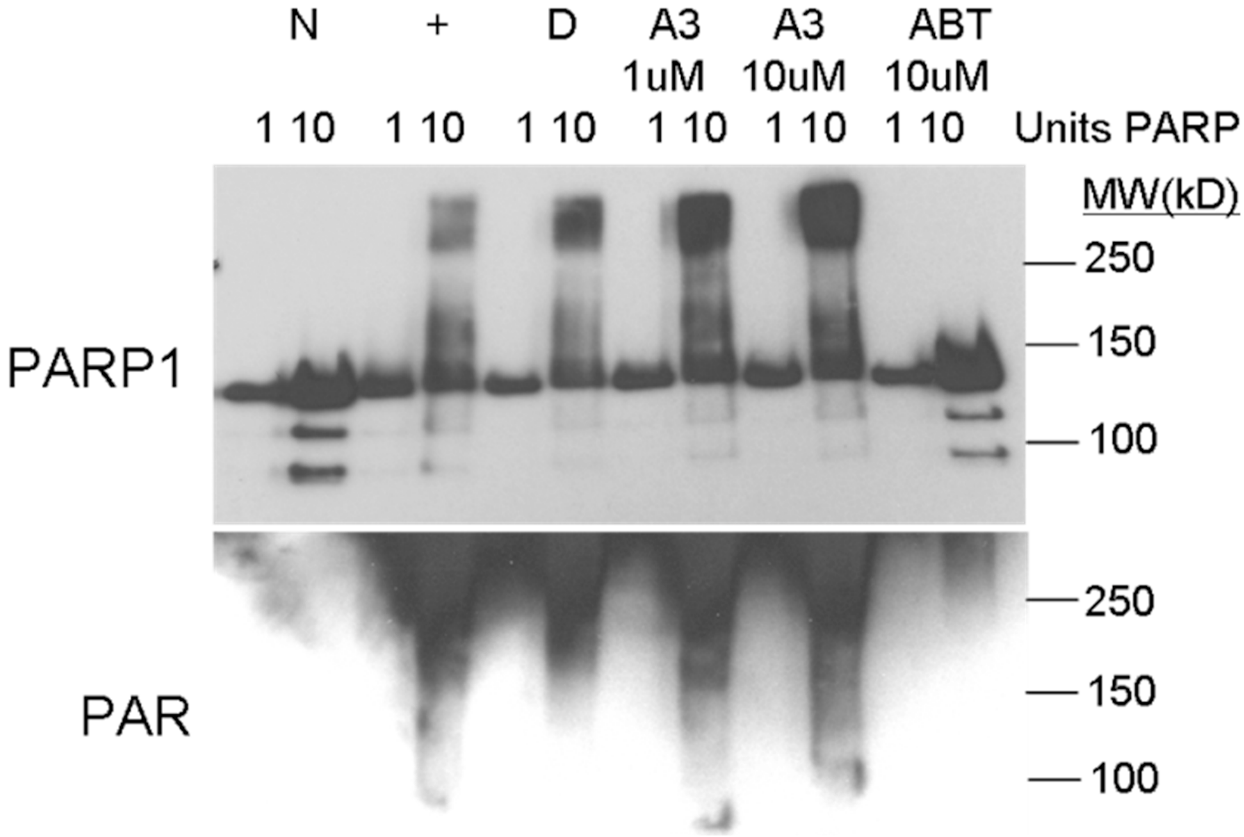

Supplementary Figure S4: *In vitro* parylation assay - *In vitro* auto-Parylation assay reveals no effect of A3 on PARP1 enzymatic activity, ABT-888 (ABT) fully inhibits PARP1 in a parallel experiment. N = neg ctl (no NAD+)+ = complete rxn, D = DMSO 2% final A3 = at 1 uM or 10 uM in 100% DMSO, to 2% DMSO final, ABT = ABT-888 (Veliparib) at 10 uM in 100% DMSO to 2% final.

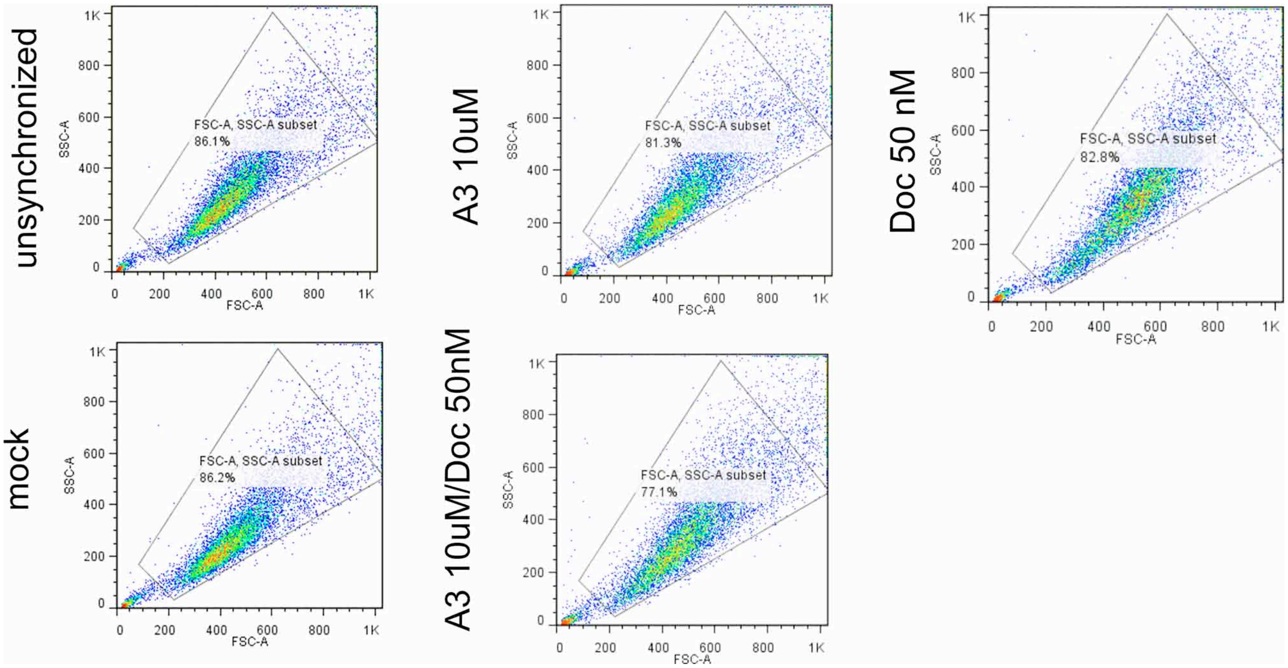

Supplementary Figure S5: Forward/sideward scatter analysis of the experiment in Figure 3H demonstrates no significant cell death after docetaxel and 'A3' exposure.
